# OP45 Adapting To Change – Horizon Scanning For New And Repurposed Medicines For Health Technology Assessment In The UK

**DOI:** 10.1017/S0266462325101037

**Published:** 2025-12-29

**Authors:** Sarah Khan, Sonia Garcia Gonzalez-Moral, Ross Fairbairn, Rhiannon Potter, Alex Inskip, Amy Hussain, Gill Norman

## Abstract

**Introduction:**

The Innovation Observatory (IO) uses horizon scanning (HS) for systematic identification of emerging medicines for the health technology assessment (HTA) process of the National Institute for Health and Care Excellence (NICE) in the UK. The IO’s HS methods have evolved with the policy and demands of HTA for medicines. We reported the evolution of HS processes to facilitate NICE’s HTA process.

**Methods:**

The remit applied by the IO to identify relevant information has evolved to become inclusive of trials in Australia, Japan, New Zealand, and Singapore to accommodate changes in regulatory procedures in the UK. Novel processes have been introduced to gather information from additional sources since inception of the IO in 2017. Semi-automation has been introduced alongside manual processes to gather, sift, and triangulate intelligence from several sources, to then populate a bespoke internal database called the Medicines Innovation Database (MInD). Additionally, a bespoke live, confidential, and searchable dashboard was developed in 2019 to improve collaboration with NICE.

**Results:**

Initially, information was shared with NICE via an early awareness form, a filtration form, and then a technology briefing (TB). Since 2019, NICE receives monthly TBs along with a live dashboard. TBs are sent to NICE 24 months from estimated approval in the UK, which is the first step in the HTA process. Since inception of the IO in April 2017, over 1,200 TBs have been submitted to NICE to kickstart the HTA process. The live dashboard provides NICE with real-time information on medicines on record being monitored in MInD, allowing better transparency, efficiency, and resource planning.

**Conclusions:**

HS systems need to be systematic and agile, to allow adaptation to the needs of HTA stakeholders for timely decision-making regarding emerging medicines that might reach the market. The IO HS methods are a potential blueprint for constructing a robust and flexible early awareness system using HS to support HTA processes. Progressive additional automation will be supported imminently.

